# CircPVT1 weakens miR-33a-5p unleashing the c-MYC/GLS1 metabolic axis in breast cancer

**DOI:** 10.1186/s13046-025-03355-1

**Published:** 2025-03-20

**Authors:** Alina Catalina Palcau, Claudio Pulito, Valentina De Pascale, Luca Casadei, Mariacristina Valerio, Andrea Sacconi, Valeria Canu, Daniela Rutigliano, Sara Donzelli, Federica Lo Sardo, Francesca Romana Auciello, Fulvia Pimpinelli, Paola Muti, Claudio Botti, Sabrina Strano, Giovanni Blandino

**Affiliations:** 1 Microbiology and Virology Unit, San Gallicano Dermatological Institute IRCSS, Rome, 00144 Italy; 2 Translational Oncology Research Unit, IRCCS, Regina Elena National Cancer Institute, Rome, Italy; 3 Department of Chemistry, “Sapienza” University of Rome, Piazzale A. Moro 2, Rome, 00185 Italy; 4 VLC Biochem Solutions, Chieti, 66100 Italy; 5 Clinical Trial Center, Biostatistics and Bioinformatics, IRCCS Regina Elena National Cancer Institute, Rome, 00144 Italy; 6 Department of Health Research Methods, Evidence, and Impact, Faculty of Health Sciences, McMaster University, Hamilton, ON Canada; 7 Department of Biomedical, Surgical and Dental Sciences, University of Milan, Milan, Italy; 8 Department of Surgery, IRCCS, Regina Elena National Cancer Institute, Rome, 00144 Italy; 9 SAFU Laboratory, IRCCS Regina Elena National Cancer Institute, Rome, Italy

**Keywords:** Non-coding RNAs, Metabolism, Breast cancer, MYC, Patients derived organoids

## Abstract

**Background:**

Altered metabolism is one of the cancer hallmarks. The role of circRNAs in cancer metabolism is poorly studied. Specifically, the impact of circPVT1, a well-known oncogenic circRNA on triple negative breast cancer metabolism is mechanistically underexplored.

**Methods:**

The clinical significance of circPVT1 expression levels was assessed in human breast cancer samples using digital PCR and the cancer genome atlas (TCGA) dataset. The oncogenic activity of circPVT1 was assessed in TNBC cell lines and in MCF-10 A breast cell line by either ectopic expression or depletion of circPVT1 molecule. CircPVT1 mediated metabolic perturbation was assessed by 1 H-NMR spectroscopy metabolic profiling. The binding of circPVT1 to miR-33a-5p and c-Myc recruitment onto the Glutaminase gene promoter were assessed by RNA immunoprecipitation and chromatin immunoprecipitation assays, respectively. The circPVT1/miR-33a-5p/Myc/GLS1 axis was functionally validated in breast cancer patients derived organoids. The viability of 2D and PDO cell models was assessed by ATP light assay and Opera Phenix plus high content screening.

**Results:**

We initially found that the expression of circPVT1 was significantly higher in tumoral tissues than in non-tumoral breast tissues. Basal like breast cancer patients with higher levels of circPVT1 exhibited shorter disease-free survival compared to those with lower expression. CircPVT1 ectopic expression rendered fully transformed MCF-10 A immortalized breast cells and increased tumorigenicity of TNBC cell lines. Depletion of endogenous circPVT1 reduced tumorigenicity of SUM-159PT and MDA-MB-468 cells. 1 H-NMR spectroscopy metabolic profiling of circPVT1 depleted breast cancer cell lines revealed reduced glycolysis and glutaminolitic fluxes. Conversely, MCF-10 A cells stably overexpressing circPVT1 exhibited increased glutaminolysis. Mechanistically, circPVT1 sponges miR-33a-5p, a well know metabolic microRNA, which in turn releases c-MYC activity promoting transcriptionally glutaminase. This activity facilitates the conversion of glutamine to glutamate. CircPVT1 depletion synergizes with GLS1 inhibitors BPTES or CB839 to reduce cell viability of breast cancer cell lines and breast cancer-derived organoids.

**Conclusions:**

In aggregate, our findings unveil the circPVT1/miR-33a-5p/Myc/GLS1 axis as a pro-tumorigenic metabolic event sustaining breast cancer transformation with potential therapeutic implications.

**Supplementary Information:**

The online version contains supplementary material available at 10.1186/s13046-025-03355-1.

## Introduction

Breast cancer is one of the most frequent causes of death among women worldwide. GLOBOCAN estimated that female breast cancer overcame lung cancer as the leading cause of global cancer incidence in 2020 with 2.3 million new cases [1]. Breast cancer is a rather heterogeneous type of tumor that can be molecularly classified in four different subtypes: Luminal A, Luminal B, HER2-positive and triple negative breast cancers (TNBC). Among the different subtypes, triple negative breast cancer subtype that expresses no oestrogen, progesterone and human epidermal growth factor receptor 2 is the most aggressive one with the poorest prognosis. Indeed, in the absence of a specific target therapy, the common treatment is based either on chemotherapy or radiotherapy [2]. In addition, in 2021 FDA approved Pembrolizumab for treatment of patients with early TNBC [3].

CircRNAs have been thought to be generated by splicing errors, but they exert important roles within non-tumoral and tumoral cells. These RNAs molecules due to their covalently closed structure are very resistant in body fluids where they can be evaluated through liquid biopsy [4]. Indeed, their expression it has been demonstrated to be altered in tumors such as breast cancer, in which they can either prevent or trigger tumorigenesis [1, 5, 6]. Circular RNAs function as scaffolds, as they interact directly with proteins or also be translated in a cap-independent mechanism generating functional peptides [7]. The most studied and debated mechanism of circRNA function is the miRNAs sponge activity. Interestingly, circRNAs roles in cancer have been deeply investigated since several studies reported their oncogenic or tumor suppressor activity [8]. In particular, Cao et al., demonstrated that circRNF20 promotes breast cancer tumorigenesis though the sponging of miR-487a [9] while Fu B. and colleagues highlighted a pivotal role of circBCBM1 in promoting breast cancer brain metastasis by regulating the expression of miR-125a [5]. Further investigation of the mechanism through which circRNAs are involved in tumorigenesis could be promising for future therapy treatments as in particular for triple negative breast cancer (TNBC) considering the limited presence of therapeutic targets.

miRNAs are small sequences of 22–24 nucleotides playing an important role in the regulation of gene expression by binding to the 3’UTR of their mRNA targets [10]. Circulating miRNAs seem to be promising biomarkers for diagnosis and prognosis of diseases including cancer [11, 12]. miRNAs have been proposed as potential biomarkers since their expression levels can be measured also in body fluids either to evaluate the progress of the therapy or even to instruct toward a specific therapy [13]. CircPVT1 originates from the circularization of exon 2 of PVT1 gene, located near MYC locus, that encodes also for a lncRNA called lncPVT1 [14, 15]. CircPVT1 exerts oncogenic activities in several tumors including gastric cancer or head and neck squamous cell carcinoma [16, 17]. Altered metabolism is one of the pivotal hallmarks of cancer [18, 19]. Cancer cells require high amount of energy in terms of ATP to sustain their growth but also to obtain precursors of important molecules. Several metabolic pathways become altered during transformation including glycolysis and glutaminolysis. Glutamine serves as a source of reduced nitrogen for biosynthetic reactions and as source of carbon for molecules biosynthesis [20]. Glutaminase, the key enzyme of glutaminolysis converts glutamine into glutamate which in turn is converted in α-ketoglutarate (α-KG) by glutamate dehydrogenase-1 (GLUD1), allowing the entrance in the tricarboxylic acid (TCA) cycle. Recently, altered glutamine metabolism has been associated with TNBC progression and metastasis [21].

In the present study, we found that aberrantly expressed circPVT1 sponges miR-33a-5p and unleashes MYC/GLS metabolic axis sustaining altered glutaminolysis. Interestingly, the treatment of triple negative breast cancer-derived organoids with GLS1 inhibitors such as CB839 and BPTES strongly reduced cell viability. Altogether these findings with those documenting that circPVT1 altered expression in breast cancer patients associates with poor overall survival, identify an unexplored network which might hold therapeutic potential.

## Methods

### Cell culture and transfection

Human breast cancer cell lines SUM-159PT, MDA-MB-468 and normal human breast epithelial cell line MCF-10 A were purchased from the American Type Culture Collection (ATCC, Manassas, VA, USA). SUM-159PT and MDA-MB-468 cells were grown in DMEM/F12 Glutamax medium (Invitrogen, Carlsbad, CA, USA) supplemented with 10% fetal bovine serum, 100 units/mL Pen/Strep antibiotic and Insulin 5 μg ml − 1 (Sigma, Saint Louis, USA) at 37 °C in a balanced air humidified incubator with 5% CO2. MCF-10 A cells were grown in DMEM/F12 Glutamax (Invitrogen, Carlsbad, CA, USA) supplemented with 10% horse serum and 100 μL of EGF 20 ng/ml, 500 μL of Antibiotic 100X, 500 μL of HC 500 ng/ml and 500 μL of Human insulin 0.01 mg/ml at 37 °C in a balanced air humidified incubator with 5% CO2.

Lipofectamine RNAimax (Invitrogen, Carlsbad, CA, USA) was used in accordance with the manufacturer’s instruction for transfection with siRNAs and miRNA mimics. SiRNAs were used at the final amount of 300 pmol in 100 mm dish. si-circPVT1 5’-CUUGAGGCCUGAUCUUUUA-3’ was used for functional in vitro experiments. For mature miR-33a-5p overexpression, we used the mirVana miR-33a-5p mimic (Ambion) at a final concentration of 5 nM and as control we used the mirVana miRNA mimic, Negative Control #1 (Ambion), at the same concentration. The circPVT1 overexpression in MCF-10 A cells was performed using 4 μg pcDNA3-circPVT1 [17] and 4 μg pcDNA3 vector as control. Plasmids were transfected with Lipofectamine 2000 (Invitrogen Carlsbad, CA, USA) in accordance with the manufacturer’s instruction at a final concentration of 1 μg in a 60 mm dish. Cells were collected 48–72 h post transfection for subsequent analyses.

### RNA processing and qPCR

The total RNA was extracted with TRizol (Thermo Fisher Scientific, Rockford, IL, USA) following the manufacturer’s instructions and the concentration, purity, and quality of total RNA were assessed using a Nanodrop TM 1000 spectrophotometer (Nanodrop Technologies).

### cDNA synthesis and qRT-PCR

One microgram of total RNA was reverse transcribed at 37 °C for 60 min in the presence of random hexamers and Moloney murine leukaemia virus reverse transcriptase (Invitrogen, Carlsbad, CA, USA). Specific oligonucleotide primers for

ACTIN Fw: 5′-GGCATGGGTCAGAAGGATT-3′ and

Rv: 5′-CACACGCAGCTCATTGTAGAAG-3′

CircPVT1 Fw:5’CGACTCTTCCTGGTGAAGCATCTGAT-3’ and

Rv:3’ TACTTGAACGAAGCTCCATGCAGC-5’;

lncPVT1 Fw:5’-GAAAGGATGTTGGCG-3’

Rv: 5’-AGGGGAGATGATTCA-3’

c-MYC Fw: 5′-CTCCTGGCAAAAGGTCAGAG-3’ and

Rv: 5′-TCGGTTGTTGCTGATCTGTC-3′;

GLS Fw: 5’-TTCCAGAAGGCACAGACATGGTTG-3’ and

Rv: 5’-GCCAGTGTCGCAGCCATCAC-3’ were used for PCR analyses. Gene expression levels were measured by quantitative real-time PCR using the SYBR Green assay (Thermo Scientific, Rockford, IL, USA) on a StepOne instrument (Thermo Scientific, Rockford, IL, USA). Small amounts of RNA (10 ng) were reverse transcribed using the TaqMan microRNA Reverse Transcription Kit (Applied Biosystems) in a final volume of 10 μl using an ABI Prism 7000 Sequence Detection System (Thermo Scientific, Rockford, IL, USA). The PCR reactions were initiated with a 10-min incubation at 95 °C followed by 40 cycles of 95 °C for 15 s and 60 °C for 60 s. qPCR quantification of miRNA expression was performed using TaqMan MicroRNA® Assays (Thermo Scientific, Rockford, IL, USA) according to the manufacturer’s protocol. RNU48 and RNU44 were used as an endogenous control to normalize miRNA expression. All reactions were performed in triplicate. For circPVT1, PVT1, MYC, GAPDH and GLS1 gene expression analysis, reverse transcription and RT-qPCR were performed using MMLV RT (Invitrogen, Carlsbad, CA, USA) and SYBR Green® Assays (Thermo Scientific, Rockford, IL, USA), respectively, according to the manufacturers’ instructions.

### Digital PCR analysis

The samples were analyzed with using the Quant Studio™ Absolute Q Digital PCR chip/plate-based system (Life Technologies). Reactions were prepared in a final volume of 10 μL, including 2 μL of 5x Master Mix, 0.5 μL of 20x TaqMan® probe, 7.5 μl of template and loaded onto dPCR chips. The thermal cycles were as follows: 10 min at 96.0 °C, 39 cycles at 96 °C for 5 s and 15 s at 60 °C. FAM fluorescence threshold values were calculated automatically by ThermoFisher Software, reviewed manually, and then applied to the corresponding cDNA. Digital PCR (dPCR) uses the procedure of end-point PCR but splits the PCR reaction into many single partitions, in which the template is randomly distributed across all available partitions. After PCR, the amplification target is detected by measuring the fluorescence in all positive partitions. The total number of copies of the target molecule in all valid partitions of a well is calculated by multiplying the copies of the target molecule per partition with the number of valid partitions. We normalized the number of copies of the target molecules on the concentration of each sample and subsequently, we applied the following equation, as reported in Verduci et al., 2017, to determine the amount of circPVT1 and miR-33a-5p molecules in MCF-10 A circPVT1 overexpressing clone respect to the control MCF-10 A and in the analyzed TNBC sample tissues.

$$\:\frac{\text{c}\text{i}\text{r}\text{c}\text{P}\text{V}\text{T}1\text{n}\text{g}\text{*}6.022\text{x}1023\text{m}\text{o}\text{l}\text{e}\text{c}\text{u}\text{l}\text{e}\text{s}/\text{m}\text{o}\text{l}\text{e}}{\text{c}\text{i}\text{r}\text{c}\text{P}\text{V}\text{T}1\text{l}\text{e}\text{n}\text{g}\text{t}\text{h}\:\left(410\text{b}\text{p}\right)\text{*}1\text{x}109\text{n}\text{g}/\text{g}\text{*}660\text{g}/\text{m}\text{o}\text{l}\text{e}}$$

### PDO cultures

For organoid generation, tissues were processed as described in Sash et al., 2018 [22]. Briefly, tissues were mechanically processed until small portions (1mm3) were obtained. Then they were enzymatically digested for 1 h at 37 °C with Tumor Dissociation Kit, human (Miltenyi, Bergisch Glabach, Germany) according to the manufacturer’s instructions. The suspension was strained over a 70 μm filter. Isolated cell clusters were resuspended in Matrigel® (Corning, New York, USA) and plated in 24-well plates.

PDO were grown in Ad-DM medium supplemented described as in Donzelli S. and colleagues [23] with 1X Glutamax (Thermo Scientific, Rockford, IL, USA), 10mM Hepes (Thermo Scientific, Rockford, IL, USA), 1X Penicillin/Streptomycin (Thermo Scientific, Rockford, IL, USA), 50 μg/mL Primocin (InVivogen Toulouse, France),, 1X B27 supplement (Thermo Scientific, Rockford, IL, USA), 250 ng/mL R-spondin 1 (PeproTech, Cranbury, NJ, USA), 5 nM Heregulin β − 1 (PeproTech, Cranbury, NJ, USA), 100 ng/mL Noggin (PeproTech, Cranbury, NJ, USA), 20 ng/mL FGF-10 (PeproTech, Cranbury, NJ, USA), 5 ng/mL FGF-7(PeproTech, Cranbury, NJ, USA), 5 ng/mL EGF (PeproTech, Cranbury, NJ, USA), 5 μM A83-01 (Tocris Bioscience), and 500 nM SB202190 (PeproTech, Cranbury, NJ, USA). Moreover, 5 μM Y-27,632 (PeproTech, Cranbury, NJ, USA) was added to culture media for the first three days of culture. When confluency was reached, organoids were dissociated by resuspension in 2 mL TrypLE Express (Invitrogen Carlsbad, CA, USA) and incubation for 10 min at room temperature. After enzyme neutralization and washing, organoids were resuspended in Matrigel and reseeded as above in order to allow the formation of new organoids. Bright-field imaging of organoids was performed on an NEXCOPE microscope. The consent for the generation of organoids derived from breast cancer patients was approved by the institutional review board of Regina Elena National Cancer Institute and appropriate regulatory authorities (approval no. IFO 1270/19). All patients signed an informed consent.

### Cell and PDOs viability assay

Viability of treated cells and PDOs was assessed using ATPlite assay (Perkin Elmer, Massachusset, USA) accordingly to the manufacturer’s instructions. Cells (8 × 102cells) and PDOs were seeded in 96 well-plates and cultured for 24 h and treated for 72 h with BPTES or CB839. Each plate was evaluated immediately on a microplate reader (EnSpire Technology, Perkin Elmer, Massachusetts, USA).

### Opera Phenix plus imaging

The PDOs were dissociated in 2 ml of TrypleE (Invitrogen 12605036), incubated at 37° for 10 min. After digestion, 10mL of medium is added and cells were centrifugated for 5 min at 1000 rpm. The dissociated PDOs were, then, resuspended in 700uL of Matrigel and 1000 cells per well were seeded (Matribot, Corning) in a PhenoPlate 96-well, black, optically clear flat-bottom, 96 wells (Revvity, Waltham, Massachusets, USA). After drops solidification, 100 μl of medium is added. When PDOs reached an area dimension of more than 500 μm2 they were treated. Number, area, perimeter, width, length and cytotox staining (Incucyte Cytotox Green Dye, Sartorius) of the spheroids were assessed by using the Opera Phenix® Plus high throughput microplate confocal imager (Revvity) and calculated by using the Harmony High-Content Imaging and Analysis Software (Revvity).

### Clonogenic assay

Transfected BC cells at a density of 1,000 were seeded in six-well plates. Cell colonies were subsequently washed, fixed, and stained until the colonies were visible. Then, colonies were counted and imaged.

### Transwell invasion assay

Transfected BC cells were added into the upper chamber with 200 μL of serum-free medium. After culturing for 24 h for the SUM-159PT, MDA-MB-468 and MCF-10 A cell lines, cells that migrated to the opposite side of the filter were fixed, stained, imaged (Leica Microsystems, Germany), and counted.

### Protein extracts and western blot analysis

Cells were homogenized on ice for 30 min in a lysis buffer composed by 50 mM, Hepes pH 7.5, 5 mM EDTA pH 8.0, 10 mM MgCl2, 150 mM NaCl, 50 mM NaF, 20 mM β-glicerophosphate, 0.5% NP40, 0.1 mM sodium orthovanadate, 1 mM PMSF, 1 mM dithiothreitol (DTT), and protease inhibitor cocktail (Roche). Lysates were clarified by centrifugation for 10 min, max speed, at 4 °C. Proteins (30 μg/lane) were separated on 10% SDS-polyacrylamide gels and transferred to nitrocellulose membranes. Immunoblots were probed with the following primary antibodies: rabbit monoclonal anti-c-Myc (DO1; Oncogene Science Uniondale, NY, USA), and mouse monoclonal anti-GAPDH (Calbiochem). Immunostained bands were detected by a chemiluminescent UVITEC Alliance 4.7 instrument (Cambridge, UK). ECL solution (entry-level peroxidase substrate for enhance chemiluminescence) (Thermo Scientific, Rockford, IL, USA) was loaded on the membrane in order to allow the chemiluminescent reaction between horseradish peroxidase (HRP) labelled on the secondary antibody and the peroxidase substrate of ECL solution. The reaction generates energy that is released in the form of light and in this way the protein signal is detected by the camera.

### Chromatin immunoprecipitation assay (ChIP)

ChIP Assay Kit (Millipore, Bedford, MA) was used according to manufacturer’s instructions. In brief, the 1% formaldehyde cross-linked chromatin was sonicated into fragments and then immunoprecipitated using MYC and POL2(ser5p) antibodies. IgG was used as negative control. DNA fraction was analyzed by qRT-PCR.

### MagIC beads RNA pull down

Samples of total, unfragmented RNA from SUM-159PT and MCF-10 A #7 cells were incubated with MagIC Beads targeting human circPVT1 transcript according to manufacturer’s instructions (https://elementzero.bio/magic-beads-rna-enrichment/). RNA attached to the beads was washed, eluted and subjected to cDNA synthesis. Levels of miR-33a-5p, miR-145 and miR-203 were measured in the input and enriched samples with RT-qPCR.

### Subcellular fractionation

Nuclear and cytoplasmic extraction reagents (Thermo Fisher Scientific, Rockford, IL, USA) were used for subcellular fractionation of BC cells. We used H3 as the nuclear control and Tubulin as the cytoplasmic control.

### Seahorse analysis

Analyses of Glutamine Dependency were performed on live cells using a Seahorse XF HS Mini Bioanalyser (Agilent Technologies; Santa Clara, CA, USA) and the Mito Fuel Flex Test (Agilent Technologies; Santa Clara, CA, USA). SUM-159PT and MCF-10 A #7 cells overexpressing miR-33a-5p (4 × 10^4^ cells/well) were seeded and grown over night prior to analysis. Assays were performed using protocols suggested by the manufacturer (Agilent Technologies; Santa Clara, CA, USA). Briefly, after a 30-min calibration of the XF sensor with a preincubated sensor cartridge, the cell plate was loaded into the analyzer, and Oxygen Consumption Rate (OCR) was analyzed under basal conditions. Glutamine Dependency was tested by first injecting BPTES (3 μM), an inhibitor of allosteric GLS1 glutaminase, followed by inhibition of carnitine palmitoyl-transferase 1 A (CPT1A) and mitochondrial pyruvate transporter (MPC) using Etomoxir (4 μM) and UK5099 (2 μM) respectively. OCR was measured after BPTES injection and following the injection of the other two inhibitors (All Inhibitors). After performing MFFT assays, cells were stained with DAPI and the cell count of each well was determined by imaging the cells using Cell Profiler. Once the assay was normalized on cell number, Glutamine Dependency was calculated with the following equation:

$$ {\rm{Dependency\% }}\,{\rm{ = }}\,{\rm{100*}}\left[ {\frac{{{\rm{Baseline}}\,{\rm{OCR - Glutamine}}\,{\rm{Inhibitor}}\,{\rm{OCR}}}}{{{\rm{Baseline}}\,{\rm{OCR - All}}\,{\rm{Inhibitors}}\,{\rm{OCR}}}}} \right] $$

### ^1^H-NMR spectroscopy

All 2D 1 H J-resolved (JRES) NMR spectra were acquired on a 500 MHz VNMRS Varian/Agilent spectrometer (Agilent, Santa Clara, CA) at 25 °C using a double spin echo sequence with pre-saturation for water suppression and 16 transients per increment for a total of 32 increments. These were collected into 16 k data points using spectral widths of 8 kHz in F2 and 64 Hz in F1. Each free induction decay (FID) was Fourier transformed after a multiplication with sine-bell window functions in both dimensions. JRES spectra were tilted by 45°, symmetrized about F1, referenced to lactic acid at δH = 1.33 ppm and the proton-decoupled skyline projections (p-JRES) exported using Agilent VNMRJ 3.2 software. The exported p-JRES were aligned, corrected for baseline offset and then reduced into spectral bins with widths ranging from 0.02 to 0.06 ppm by using the ACD intelligent bucketing method (1D NMR Manager software, ACD/Labs, Toronto, Canada). This method sets the bucket divisions at local minima (within the spectra) to ensure that each resonance is in the same bin throughout all spectra. The area within each spectral bin was integrated and in order to compare the spectra, the integrals derived from the bucketing procedure were normalized to the total integral region. Metabolites were identified using an in-house NMR database and literature data and confirmed by 2D homo- and hetero-nuclear NMR spectroscopy.

### NMR spectra pre-processing treatment

The 1D skyline projections exported were aligned and then reduced into spectral bins with ranging from 0.01 to 0.02 ppm by using the ACD intelligent bucketing method (1D NMR Manager software (ACD/Labs, Toronto, Canada). To compare the spectra, the integrals derived from the binning procedure were normalized to the total integral region, following exclusion of bins representing the residual water peak (4.33–5.17 ppm) and the TSP peak (0.5–0.5 ppm). The resulting data was used as input for multivariate analysis: Principal Component Analysis (PCA and Orthogonal projections to latent structures discriminant analysis (OPLS-DA) were performed using SIMCA-*P* + version 12 (Umetrics, Umea, Sweden).

### TCGA analysis

Normalized gene, miRNA, and exon expression data for breast cancer (BRCA) patients were obtained from the Broad Institute TCGA Genome Data Analysis Center (Broad GDAC Firehose, 2016_01_28 run). Survival analysis was performed using the Kaplan-Meier method, with a log-rank test applied to assess the statistical significance of differences between survival curves. Clinical annotations and mutational status data were retrieved from cBioPortal (https://www.cbioportal.org/). Differences between patient subgroups were evaluated using the Wilcoxon test. Pearson’s correlation coefficient was calculated for correlation analysis. All analyses were conducted using MATLAB R2023b.

### Analysis of GLS promoter

Lasagna 2.0 web-tool to analyse GLS promoter. The promoter sequences are related to the human genome GRch38/hg38.

### Statistical analysis

The resulting data were used as input for univariate and multivariate analysis PCA and OPLS-DA. PCA and OPLS-DA were conducted using SIMCA-*P* + version 12 (Umetrics, Umea, Sweden). For microRNA analysis, Pearson’s correlation coefficient was calculated to assess quality of replicates. Generally, Student’s *t*-test was used to assess significance of the data and *P*-values ≤ 0.05 were considered statistically significant.

## Results

### Aberrant expression of circPVT1 elicits pro-tumorigenic effects in breast cancer cells

**Fig. 1:**
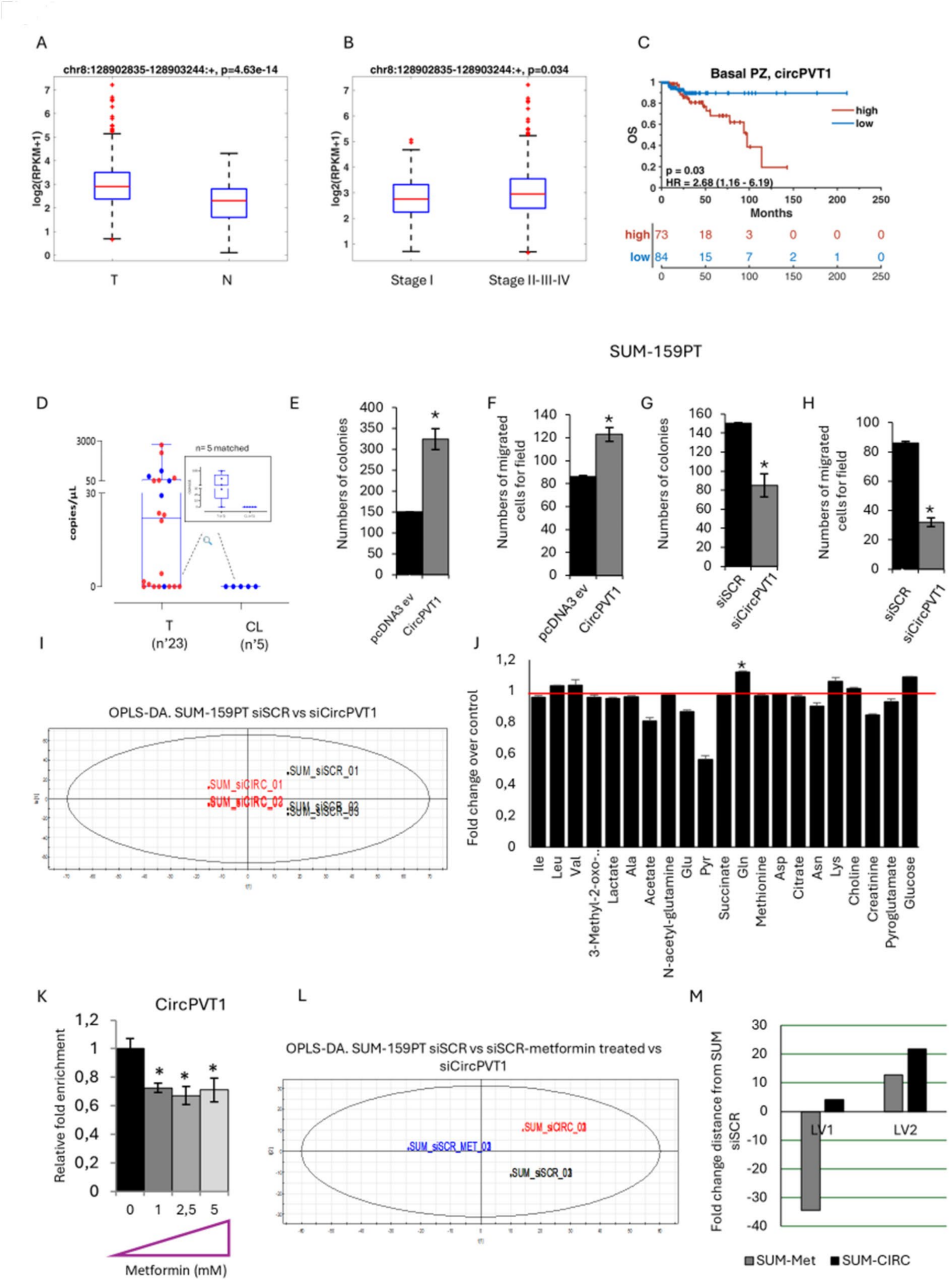
Aberrant expression of circPVT1 elicits pro-tumorigenic effects in breast cancer cells. (**A**-**B**) Log2 expression levels of chromosome interval containing circPVT1 in tumor and non-tumoral samples (**A**) and in stage I and II-III-IV tumor samples (**B**). (**C**) Kaplan Meier curves indicating the overall survival of patients basing on the expression level of circPVT1. (**D**) Boxplots show the copies/μl of circPVT1 in 23 TNBC tumors and in 5 controlateral breast tissues. Highlighted the box plot representing the 5 breast tissue tumors and their corresponding contralateral ones (blu dots). (**E**, **G**) Histograms show the number of colonies of SUM-159PT cells either expressing high (**E**) or low levels (**G**) of circPVT1. (**F**, **H**) Histograms show the number of migrated SUM-159PT cells treated as in **E**-**G**. (**I**) PCA models built on the ^1^H-NMR dataset of media samples cell extracts from SUM-159PT cell cultures either expressing endogenous or low levels of circPVT1. (**J**) Histograms show the fold changes of the most discriminant metabolites between the two groups from the PCA models (**I**). (**K**) Relative fold enrichment of circPVT1 levels measured in SUM-159PT cells treated with increased concentration of Metformin. (**L**) PCA models built on the ^1^H-NMR dataset of media samples cell extracts from SUM-159PT cell cultures either expressing endogenous levels or silenced for circPVT1 and treated or not with 0.5 mM of metformin. (**M**) *t*-test applied on LV1 and LV2 component on SUM_siSCR treated with metformin and SUM_sicircPVT

We found that circPVT1 expression was significantly higher in breast cancer patient tissues when compared with non-tumoral ones in The Cancer Genome Atlas (TCGA) data set (Fig. 1A). CircPVT1 aberrant expression was more pronounced in advanced stages (II/III/IV) compared to I stage (Fig. 1B). Interestingly, basal like breast cancer patients expressing higher levels of circPVT1 exhibit shorter overall free survival (Fig. 1C). We conducted association analyses with the two major mutated genes (TP53 and PIK3CA) in triple negative breast cancers (TNBC). No significant association was observed between *TP53* mutation status and *circPVT1* expression in both the overall breast cancer cohort and the triple-negative breast cancer subtype (Fig. S1A-B). In contrast, *PIK3CA* mutations showed a weak association with *circPVT1* expression in the entire breast cancer cohort but not in TNBC subtype (Fig. S1C-D). We also looked at PTEN, a known tumor suppressor gene whose inactivation is involved in the resistance of TNBC to anticancer treatment [24]. Both *PTEN* genomic alterations and its gene expression levels were evaluated. High *circPVT1* expression was associated with patients lacking *PTEN* genomic alterations and with low *PTEN* expression levels in the overall breast cancer cohort, but such associations were not observed in TNBC (Fig. S1E-G). Digital PCR (dPCR) analysis using specific primers spanning the junction of circPVT1 circularization revealed that circPVT1 expression was higher in breast cancer tissues than in matched non-tumoral tissues derived from patients enrolled consecutively at the IRCCS Regina Elena National Cancer Institute (Fig. 1D and Fig. S1H). To ascertain further circPVT1 oncogenic role in breast cancer, we found that overexpression of circPVT1 promoted colony formation ability and migration of SUM-159PT and MDA-MD-468 breast cancer cell lines (Fig. 1E-F) (Fig. S2A-B). Conversely, depletion of circPVT1 caused a reduction of colony formation and migration of both SUM-159PT and MDA-MD-468 cells (Fig. 1G-H) (Fig. S2A-B). This effect appears to be independent of any direct effect of circPVT1 depletion on the linear PVT1 counterpart in SUM-159PT cells (Fig. S2C). To evaluate metabolic changes linked to the circPVT1 activity, we conducted ^1^H-NMR spectroscopy metabolic profiling of culture media of circPVT1 depleted SUM-159PT cells (SUM_sicircPVT1) compared to that of control cells (SUM_siSCR). Firstly, ^1^H-NMR data were analysed by using unsupervised PCA analysis (data not shown). Secondly, the same ^1^H-NMR dataset was analysed with the supervised method of OPLS-DA. A good predictive model (Q2 = 0.89) with one predictive and sic. orthogonal LVs R2X = 76% and R2Y = 100% was obtained (Fig. 1I). A *t*-test applied on the predicted LV1 confirmed significant metabolic difference between SUM_sicircPVT1 and SUM_siSCR cells (*p* < 0.00001). The metabolic differences are expressed as fold change over the control-silenced cells (Fig. 1J). By looking at the main metabolites correlated to a tumor phenotype, we observed that SUM_sicircPVT1 cells exhibited higher concentration of glucose and glutamine as well as lower concentrations of glutamate, lactate, alanine and acetate. These findings might indicate decreased glycolytic and glutaminolitic fluxes and increased oxidative metabolism, thereby suggesting that cancer cells produce less energy needed to grow and spread (Fig. S2D). To further understand whether the depletion of circPVT1 reprograms metabolism toward a less tumorigenic phenotype we compared its effect with that induced by metformin, an oral anti-diabetic drug with well-known metabolic activities that we have previously demonstrated [25, 26]. Interestingly, we found that metformin treatment downregulated circPVT1 expression in breast cancer cells (Fig. 1K). We then performed ^1^H-NMR spectroscopy metabolic profiling of culture media derived from SUM_siSCR treated with metformin and SUM_sicircPVT1 cells. SUM_siSCR untreated cells were included as control. The derived NMR profiles were compared by using OPLS-DA obtaining a good predictive model (Q2 = 0.9) with one predictive and three orthogonal LVS with R2X = 86% and R2Y = 100% (Fig. 1L). A *t*-test applied on LV1 showed statistically significant metabolic differences between SUM_siSCR treated with metformin and SUM_sicircPVT1 cells thereby highlighting a broader metabolic impact of metformin than circPVT1 depletion on breast cancer cells (Fig. 1M). Interestingly, on the LV2 we evidenced only statistically significant metabolic differences when SUM_siSCR untreated control cells were compared to both SUM_siSCR treated with metformin and SUM_sicircPVT1 cells (Fig. 1M). The loading analysis revealed that LV1 is indicative of glycolysis pathway while LV2 of the fatty acid synthesis. The quantitative OPLS-DA approach showed that the impact of circPVT1 depletion on fatty acid synthesis was greater than metformin treatment (LV2), while that on the glycolysis pathway (LV1) was more evident on metformin-treated breast cells (Fig. 1L-M). In aggregate, our findings report that aberrant expression of circPVT1 exerts pro-proliferative and pro-migratory effects sustaining altered glutaminolysis.

### Ectopic expression of circPVT1 promotes glutaminolysis in the non-tumorigenic MCF-10 A breast cell line

**Fig. 2:**
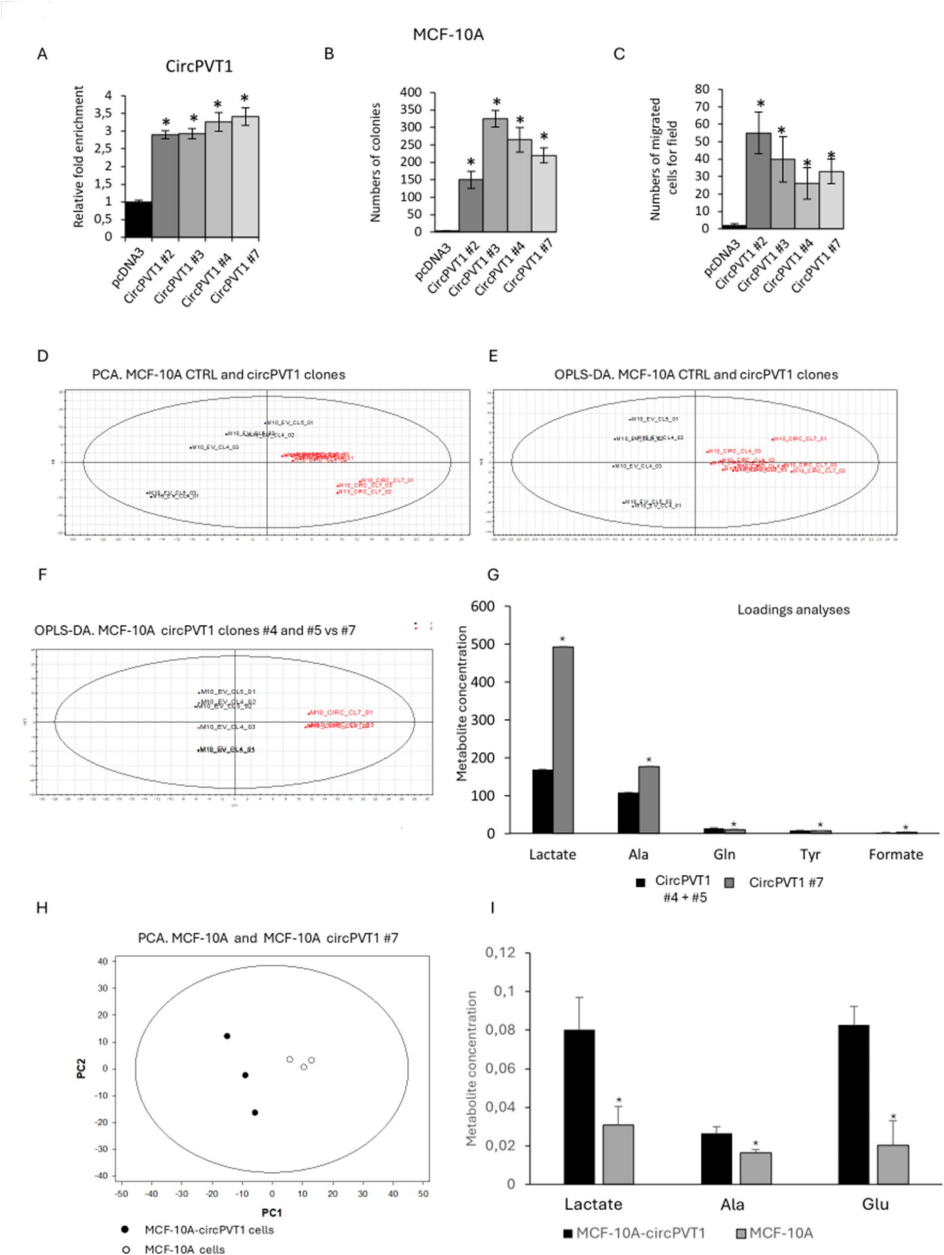
Ectopic expression of circPVT1 promotes glutaminolysis in the non-tumorigenic cell line MCF-10 A breast cell line. (**A**) CircPVT1 RNA relative enrichment levels measured in MCF-10 A stably expressing pcDNA3 or circPVT1. Numbers (#) indicated the different clones of MCF-10 A stably expressing high circPVT1 levels. (**B**-**C**) Histograms show number of colonies (**B**) or of migrated cells (**C**) count in MCF-10 A treated as in (**A**). (**D**-**E**) PCA (**D**) and OPLS-DA (**E**) models built on the ^1^H-NMR dataset of media samples cell extracts from MCF-10 A cell cultures (clones #1–7) either expressing endogenous or high levels of circPVT1. (**F**) OPLS-DA model built on the ^1^H-NMR dataset of media samples cell extracts from MCF-10 A cell clones #4,5 and 7 expressing high levels of circPVT1. (**G**) Histograms show the fold changes of the most discriminant metabolites between the different groups from the PCA model. (**H**) PCA model built on the ^1^H-NMR dataset of cellular extracts from MCF-10 A cell clone #7 either expressing endogenous or high levels of circPVT1. Legend: Black circles, MCF-10 A-circPVT1 cells; white circles, control cells. (**I**) Histograms show the fold changes of the most discriminant metabolites between the different groups from the PCA model in H

To further ascertain the pro-tumorigenic activity of circPVT1 in breast cancer development, we stably expressed circPVT1 into a non-tumorigenic breast cell line, MCF-10 A that exhibits endogenous circPVT1 levels lower than TNBC cell lines (Fig. 2A; Fig. S2E). We also found that circPVT1 overexpression did not impact on the expression of lncPVT1 (Fig. S2F-G). Intriguingly, MCF-10 A stably expressing high levels of circPVT1 were, indeed, able to form colonies and to migrate compared to control cells, thereby strengthening the oncogenic role of circPVT1 in breast cancer development (Fig. 2B-C). Subsequently, ^1^H-NMR spectroscopy metabolic profiling of culture media of three stably overexpressing circPVT1 MCF-10 A (#2, #4, #7) clones compared to control cells was performed (Fig. 2D-E). We built an OPLS-DA model containing only clone #7 (Fig. 2F). A *t*-test applied on the predictive LV1 confirmed significant metabolic differences between the two groups (*p* < 0.00001). Loading analysis revealed that the flux of the glutaminolytic pathway was increased in MCF-10 A-circPVT1#7 compared to control cells (Fig. 2G). To corroborate the results provided by the analysis of culture media, we used ^1^H-NMR spectroscopy to characterize the metabolic effects of circPVT1 overexpression in MCF-10 A cells at the intracellular level. To this end, we collected the NMR spectra of MCF-10 A circPVT1#7 clone and control cells from cellular extracts. The related data were organized accordingly to a Principal Component Analysis (PCA) model, an unsupervised method allowing orthogonal decomposition of variance associated with the analysed metabolites (Fig. 2H). A *t*-test applied on the predictive PC1 confirmed significant metabolic differences between the two groups (*p* = 0.005). The loading patterns of the most relevant metabolites on PC1 (lactate, alanine and glutamate) indicated that the flux of the glutaminolytic pathway was increased in MCF-10 A-circPVT1 compared to control cells. Indeed, MCF-10 A-circPVT1 cells showed statistically significant higher levels of lactate, alanine, and glutamate than control cells (Fig. 2I). Furthermore, the most relevant metabolite loading patterns on PC1 (lactate, alanine and glutamate) indicated the activation of alanine aminotransferase for the conversion of glutamate to alpha-ketoglutarate to enable ATP production through the truncated TCA cycle. Altogether these findings unveil an oncogenic role of circPVT1 in breast cancer which leads to altered glutaminolysis.

### CircPVT1 sponges the metabolic miR-33a-5p in breast cancer cells

**Fig. 3:**
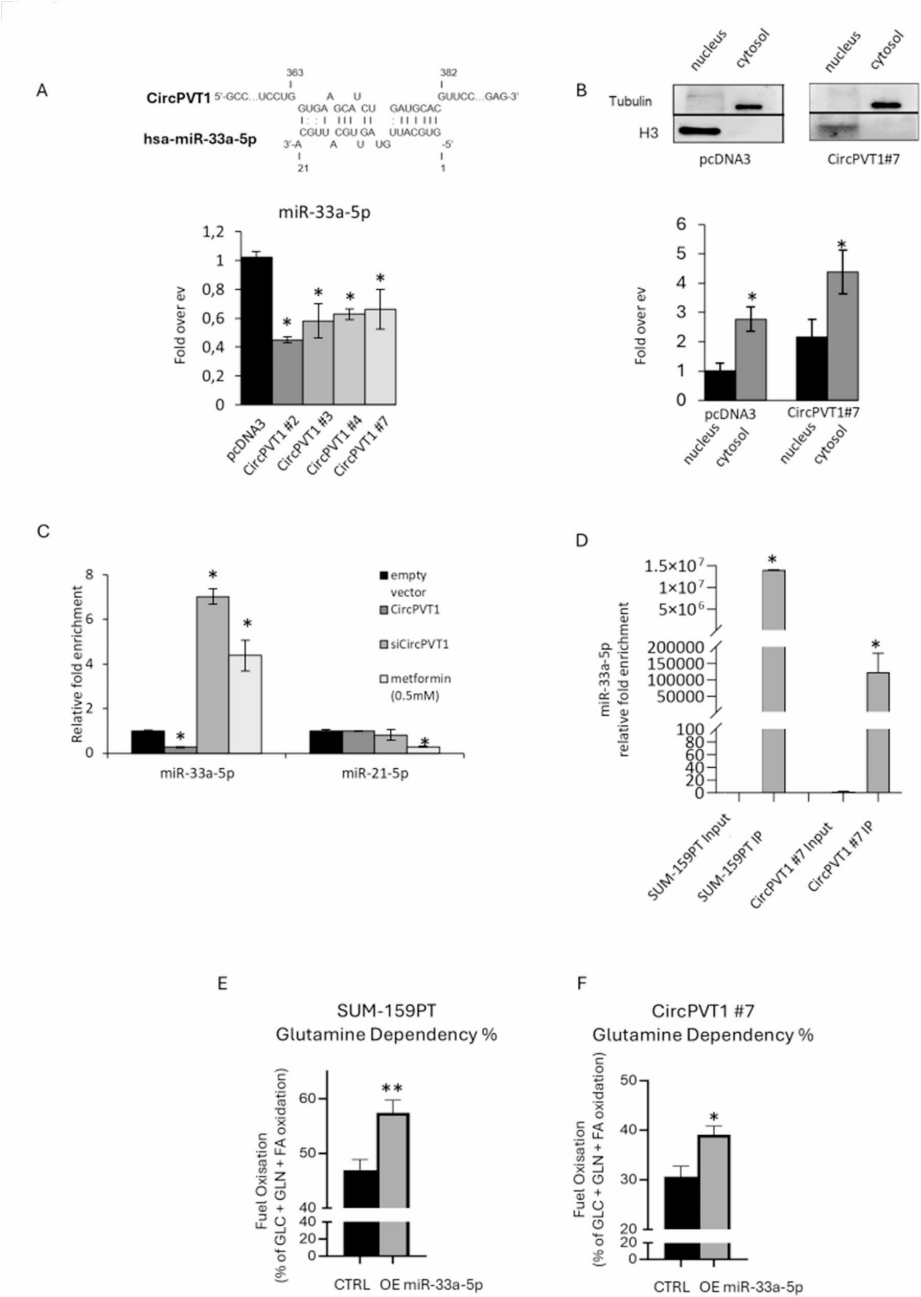
CircPVT1 sponges the metabolic miR-33a-5p in breast cancer cells. (**A**) Upper part, predictive site of binding interaction between miR-33a-5p and circPVT1. Lower part, histograms show the expression levels of miR-33a-5p in MCF-10 A cells treated as in Fig.  2A expressed in fold over empty vector (ev). (**B**) Upper part, representative protein gel blots of nucleus/cytosol cell lysates obtained from MCF-10 A cells stably expressing pcDNA3 or circPVT1 (clone #7) stained with the indicated antibodies. Lower part, histograms show the levels of circPVT1 between nucleus/cytosol obtained from MCF-10 A treated as in the upper part. (**C**) Histograms show the expression levels of miR-33a-5p and miR-21-5p measured in SUM-159PT expressing endogenous or high or low levels of circPVT1 or treated with 0.5 mM of metformin. (**D**) Histograms show the miR-33a-5p relative fold enrichment in SUM-159PT and MCF-10 A circPVT1#7 cells measured in total RNA immunoprecipitated with circPVT1-capture probes. (**E**-**F**) Histograms show the fuel oxidation rate of SUM-159PT (**E**) and MCF-10 A circPVT1#7 cells (**F**) expressing endogenous or ectopic levels of miR-33a-5p

To mechanistically decipher the contribution of altered circPVT1 expression to cancer metabolism of breast cancer cells we aimed to assess its ability to sponge microRNAs with well-established metabolic activities. Indeed, it is known that one of the major functions of circRNAs is the ability to sponge miRNAs [14]. We have previously reported that metformin-induced metabolic reprogramming of breast cancer cell lines was at least partially, mediated by the up-regulation of miR-33a-5p [27] and the downregulation of miR-21-5p [28]. Interestingly we found that circPVT1 may interact by direct binding with miR-33a-5p (Fig. 3A upper panel). Indeed, MCF-10 A-circPVT1 expressing clones exhibited reduced expression of miR-33a-5p compared to control cells (Fig. 3A lower panel; Fig. S2H). Subcellular localization of circPVT1 revealed its predominant localization in the cytoplasmic fraction (Fig. 3B). Depletion of circPVT1 in SUM-159PT cells led to increased miR-33a-5p expression (Fig. 3C). Unlike miR-33a-5p, no modulation of miR-21-5p was evidenced (Fig. 3C). Altogether these findings prompted us to assess whether circPVT1 sponges miR-33a-5p in breast cancer cells lines. RNA immunoprecipitation was performed in both SUM-159PT and in MCF-10 A clone #7 breast cancer cells. Notably, a direct biding of miR-33a-5p to circPVT1 was evidenced in both cell lines (Fig. 3D). This binding was not evidenced for miR-21-5p which was used as unrelated miRNA to test the binding specificity of circPVT1 to miR-33a-5p (Fig. S3A). Functionally, we found that ectopic expression of miR-33a-5p induced SUM-159PT and MCF-10 A/circPVT1#7 cells to use glutamine (rather than glucose and pyruvate) as preferred substratethan control cells; thereby rescuing, at least partially, the aberrant effect of circPVT1 on glutaminolysis (Fig. 3E-F). Collectively our findings originally show that circPVT1 sponging the metabolic miR-33a-5p contributes to altered glutaminolysis.

### c-MYC mediates circPVT1/miR-33a-5p-induced metabolic alteration in breast cancer cells

**Fig. 4:**
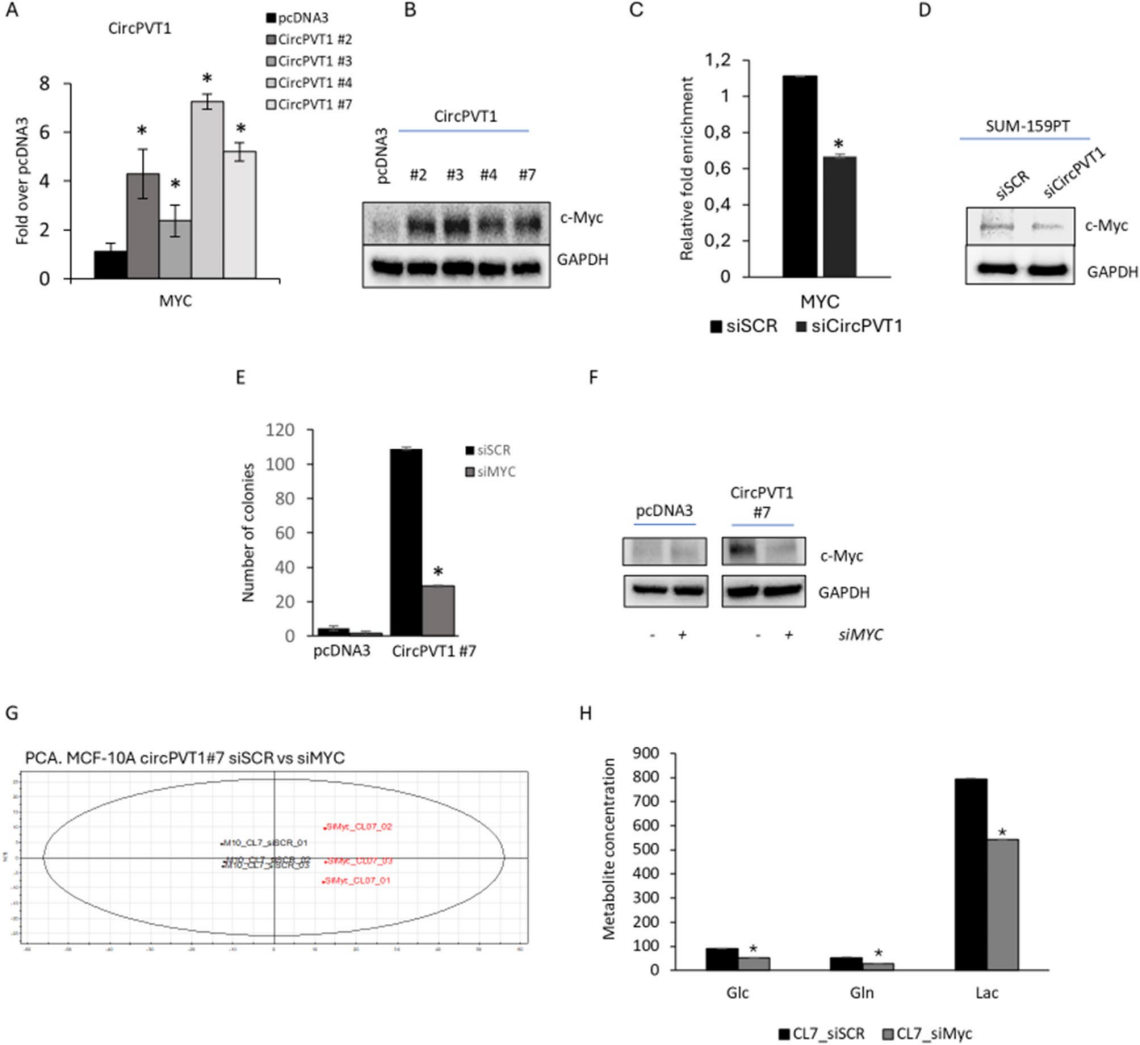
c-MYC mediates circPVT1/miR-33a-5p-induced metabolic alteration in breast cancer cells. (**A**) Histograms show the c-MYC expression levels measured in MCF-10 A treated as in Fig.  2A. (**B**) Representative protein gel blots of whole cell lysates extracted from MCF-10 A treated as in Fig.  2A. (**C**) Histograms show the c-MYC expression levels measured in SUM-159PT expressing endogenous or high or low levels of circPVT1. (**D**) Representative protein gel blots of whole cell lysates extracted from SUM-159PT cells silenced or not for circPVT1. (**E**) Histograms show the number of colonies of MCF-10 A clone #7 after c-Myc silencing. (**F**) Representative cropped protein gel blots of whole cell lysates extracted from MCF-10 A cells depleted or not for c-Myc expression (The uncropped protein gel blot is reported in Fig.S3E). (**G**) PCA models built on the ^1^H-NMR dataset of media samples cell extracts from MCF-10 A cell cultures either expressing endogenous or stably expressing high levels of circPVT1 followed silencing of c-MYC or SCR. (**H**) Histograms show the fold changes of the most discriminant metabolites between the four groups from the PCA models reported in G

To further dissect the role of circPVT1 in altered glutaminolysis of breast cancer cells we focused on c-MYC, a well-known target of miR-33a-5p known to activate at the transcriptional activity several key metabolic enzymes. Indeed, it has been reported that c-MYC fine tunes glutaminase (GLS) translation, the enzyme converting glutamine to glutamate, by directly suppressing miR-23a and miR-23b expression. This leads to aberrant GLS expression [29]. We have previously shown data that miR-33a-5p directly binds the c-Myc 3’-UTR abrogating c-MYC expression in breast cancer cells [27]. Interestingly we found a direct correlation when comparing circPVT1 and c-MYC expression levels in breast cancer TGCA database and MCF-10 A#circPVT1 expressing clones (Fig. S3B-C). Congruently, c-Myc protein expression was increased in MCF-10 A#circPVT1 expressing clones and reduced in SUM-159PT cells upon depletion of circPVT1 (Fig. 4A-D). Furthermore, ectopic uptake of miR-33a-5p mimic led to a downregulation of c-Myc both at transcriptional and protein levels (Fig. S3D). To understand the role of c-Myc in the circPVT1-mediated induction of glutaminolysis, we analysed the metabolites released in the media of MCF-10 A circPVT1 clone #7 silenced for c-MYC expression (Fig. 4E-F and Fig. S3E). The medium samples derived from the control cells (M10_siSCR clone#7) and those deriving from the c-MYC-depleted cells (si-Myc clone#7) were analysed by using ^1^H-NMR spectroscopy. OPLS-DA analysis was used to compare NMR profiles (Fig. 4G). A *t*-test applied on LV1 showed significant metabolic differences between the medium samples of the two groups (*p* < 0.00001) (Fig. 4G). Interestingly, c-MYC depleted cells exhibited increased glucose and glutamine consumption. These findings suggest increased oxidative metabolism following c-Myc depletion in MCF-10 A cells overexpressing circPVT1 (Fig. 4H).

### c-MYC regulates transcriptionally GLS1 expression

**Fig. 5:**
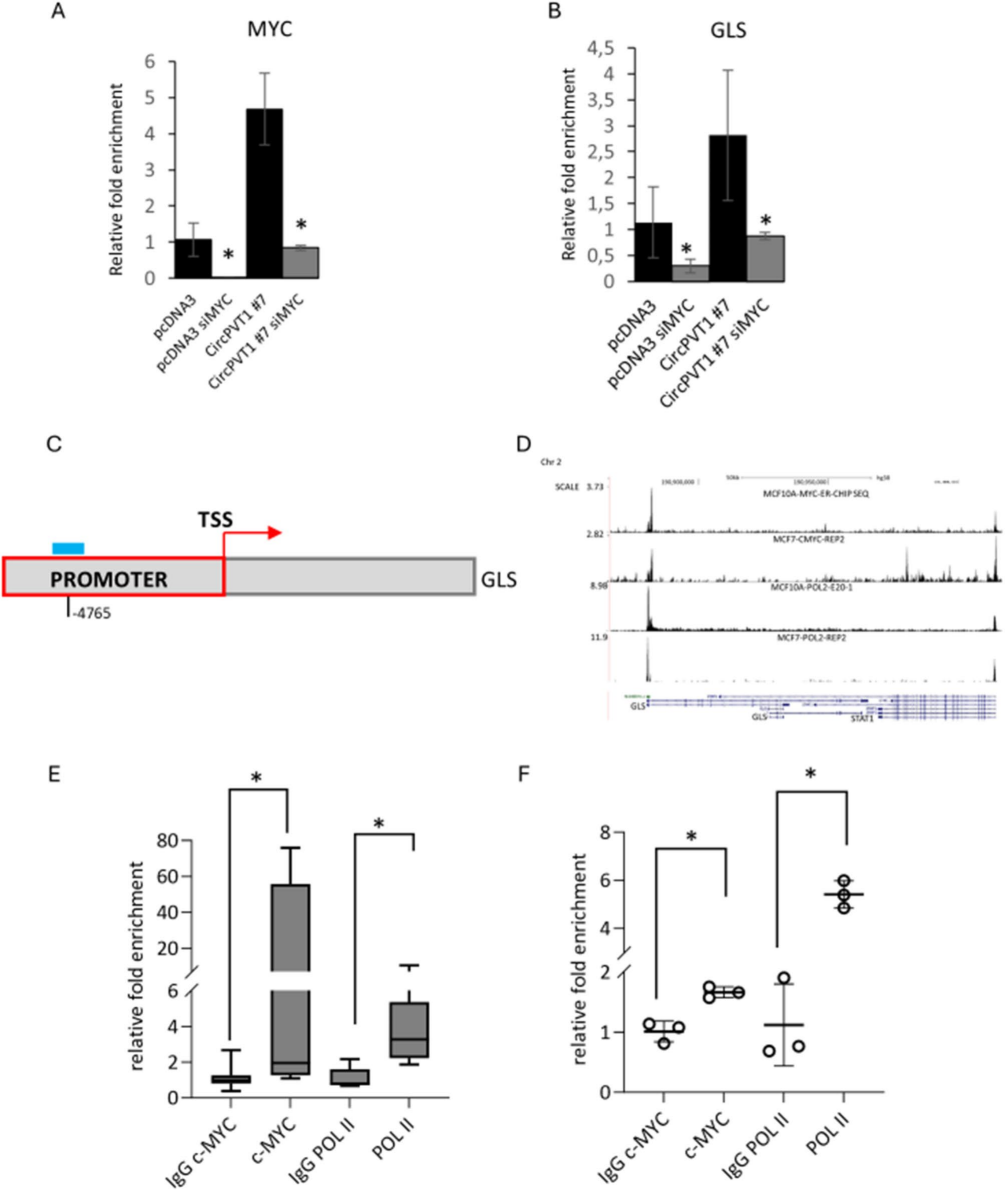
c-MYC regulates transcriptionally GLS1 expression. (**A**-**B**) Histograms show the relative c-MYC (**A**) and GLS (**B**) expression levels measured in MCF-10 A pcDNA3 and circPVT1 #7 silenced for SCR or c-MYC. (**C**) Predictive binding region of c-Myc on GLS promoter region. (**D**) c-Myc protein enrichment on GLS promoter region in MCF-10 A cells from ChIP-seq data deposited on the CistromeDataBase (http://cistrome.org/). (**E**-**F**) Relative enrichment of the occupancy of c-Myc p-Ser62 and POL II on the regulatory regions of GLS (-4850: -4811 upstream GLS transcriptional starting site) assessed by Chromatin Immunoprecipitation in MCF-10 A circPVT1 #7 (**E**) or SUM-159PT (**F**)

We aimed to assess whether c-Myc could transcriptionally regulate the expression of GLS. A positive correlation between c-MYC and GLS expression was evidenced in both breast cancer TGCA database and in MCF-10 A cell clones overexpressing circPVT1 (Fig. S3F-G). Interestingly, c-MYC depletion in cell overexpressing circPVT1 significantly reduced GLS1 transcript (Fig. 5A-B). By using Lasagna software, we identified a putative binding site for c-Myc on GLS promoter (4850 − 4811 upstream GLS transcriptional starting site) (Fig. 5C). Furthermore, the analysis of c-Myc ChIP-seq data deposited on the CistromeDataBase (http://cistrome.org/) revealed a binding of the c-Myc protein onto a specific binding site of GLS promoter region in MCF-10 A cells (Fig. 5D). Congruently, chromatin immunoprecipitation assays confirmed a direct binding of c-Myc on GLS promoter region both in SUM-159PT and in MCF-10 A clones overexpressing circPVT1 (Fig. 5E-F). The same promoter region was enriched in active Polymerase II (ser5p); thereby indicating the active transcription of GLS gene associated to the c-Myc protein binding (Fig. 5E-F). Altogether these findings support the critical role of c-Myc transcriptional activity in the aberrant regulation of GLS as downstream effector of an oncogenic signaling cascade instigated by the aberrant expression of circPVT1 in breast cancer cells.

### CircPVT1 depletion sensitizes breast cancer cells and TNBC derived organoids to the glutaminase inhibitors BPTES and CB839

**Fig. 6:**
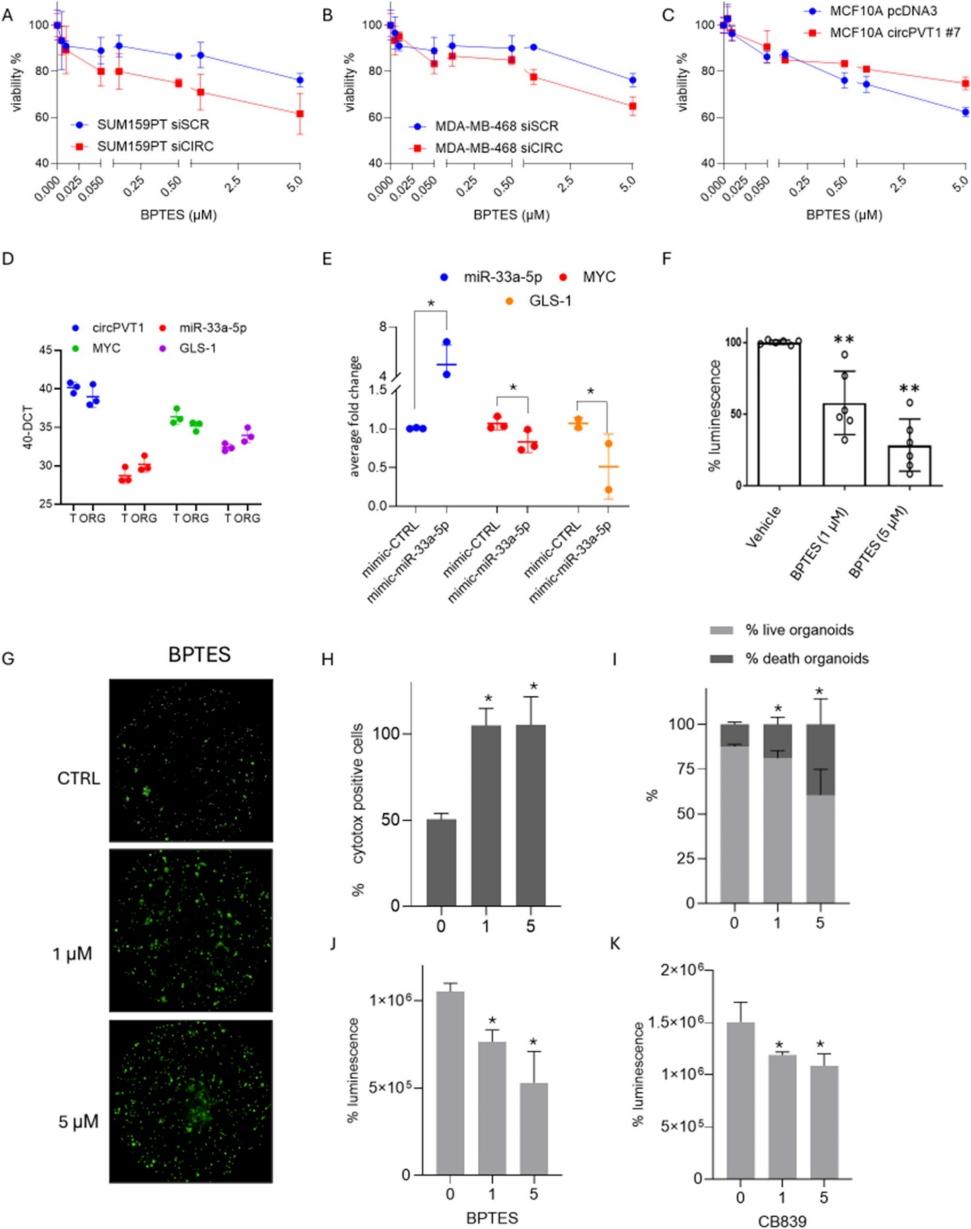
CircPVT1 depletion sensitizes breast cancer cells and TNBC derived organoids to the glutaminase inhibitors BPTES and CB839. (**A**) Viability curves obtained by measuring ATP levels in SUM-159PT cells silenced or not for circPVT1 and treated for 72 h with increasing doses of BPTES (0–5 μM). (**B**) Viability curves obtained by measuring ATP levels in MDA-MB-468 cells silenced or not for circPVT1 and treated for 72 h with increasing doses of BPTES (0–5 μM). (**C**) Viability curves obtained by measuring ATP levels in MCF-10 A circPVT1#7 and treated for 72 h with increasing doses of BPTES (0–5 μM). (**D**) Relative fold enrichment of circPVT1, miR-33a-5p, c-MYC and GLS among tumoral and matched patients derived organoids (ORG) (*n* = 3). (**E**) Relative fold enrichment of miR-33a-5p, c-MYC and GLS following miR-33a-5p overexpression. (**F**) Histograms show PDOs viability after 72 h of BPTES treatment (1 and 5 μM). (**G**) Representative images of cytotox positive cells of PDO #240 after 72 h of BPTES treatment at different doses (1 and 5 μM). (**H**) Histograms show the percentage of cytotox positive organoids after 72 h of BPTES treatment at different doses (1 and 5 μM). (**I**) Percentage of lived organoids over death organoids after #240 treatment as in G. (**J**) Percentage of luminescence of PDO #240 organoids treated as in G. (**K**) Percentage of luminescence of PDO #240 organoids after 72 h of CB839 treatment at different doses (1 and 5 μM)

Growing evidence has shown that glutamine addiction in breast cancer cells might represent a novel therapeutic target. Indeed, inhibition of glutaminase through specific inhibitors is sought to prevent aberrant cell proliferation and sensitize cancer cells to anticancer treatment. We found that siRNA-mediated depletion of circPVT1 expression rendered SUM-159PT and MDA-MB-468 more sensitive to the killing induced by BPTES and CB839 glutaminase inhibitors (Fig. 6A-B and Fig. S4A-B). Interestingly, MCF-10 A cells stably overexpressing circPVT1 are less prone to the BPTES and CB839-induced cell killing effects than control cells (Fig. 6C and Fig. S4C). Breast tumor organoids derived from three independent triple negative breast cancer patients (T3N0 grade 3, T2N3 grade 3, T4N2a grade 3) were assessed for endogenous expression of circPVT1, miR-33a-5p, MYC and GLS which resulted comparable to that of the originating breast cancer tissues (Fig. 6D). Interestingly, ectopic expression of miR-33a-5p led to downregulation of c-Myc and consequently of GLS1 expression levels, thereby providing strong evidence to the suitability of TNBC-derived PDOs to assess the response to glutaminase inhibitors (Fig. 6E). Indeed, BPTES treatment reduced in a dose-dependent manner cell viability, highlighting the critical role of glutaminase in the aberrant survival of breast cancer cells (Fig. 6F). To further assess the impact of glutaminase inhibitor on TNBC-derived PDOs we performed high-content imaging analysis on one representative PDO #240 with the aid of Opera Phenix Plus platform. This allowed evaluating the effect of BPTES at single organoid resolution. As shown in (Fig. 6G-H) the number of dead cells that show increased levels of LDH release as of indicator of cytotoxicity (cytotox positive organoid structures) increased upon BPTES treatment. Similarly, the ratio of death vs. live organoids increased, while ATP metabolic activity decreased upon BPTES treatment (Fig. 6I-J). CB839 treatment induced similar effects but to a lesser extent than BPTES (Fig. 6K).

Collectively, these findings indicate that aberrant expression of circPVT1 might contribute to glutamine addiction of breast cancer cells leading to the aberrant activation of glutaminase. Herein we also provide insights supporting the role of selective glutaminase inhibitors as anticancer therapy intercepting oncogenic networks initiated by aberrant expression of circular RNAs such as circPVT1 in breast cancer.

## Discussion

Altered metabolism is a cancer hallmark. While genetic alterations linked to cancer metabolism are the focus of intense research, the role of non-coding RNAs such as microRNAs, long non-coding RNAs and circular RNAs as initiators of cancer metabolic alterations is poorly studied. In the present manuscript we unveil the impact of circPVT1 aberrant expression on breast cancer metabolism. Indeed, we originally found that circPVT1 sponges the metabolic miRNA-33a-5p, thereby releasing aberrantly c-MYC transcriptional activity that leads to deregulated expression of glutaminase. Despite to a less extent than miR-33a-5p, we found, under the same experimental conditions, that circPVT1 sponges miR-145-5p and miR-203 (Fig. S4D-G). Being c-MYC a well-known target of all three microRNAs this might suggest that circPVT1 tightly controls c-MYC expression by sponging specific microRNAs. Both circPVT1 sponging specificity and concomitance on miR-33a-5p or other microRNAs could be dictated either by the tumoral genomic landscape or by the stage of a given tumorigenic process. There is growing evidence that metabolic alterations represent a rapid and adaptive response to oncogenic cues that might occur independently from genetic alterations. Thus, the formation of specific circRNA/microRNAs interacting codes, as herein described, might confer spatial and temporal plasticity to the metabolic adaptation of breast cancer cells.

Glutamine is a fundamental amino acid for many functions of cancer cells. It has been reported that glutamine consumption is a very active process in cancer cells when compared to that of other amino acids. TNBC subtype which lacks oestrogen receptor (ER) expression, progesterone receptor (PR) expression, Epidermal Growth factor receptor 2 (HER2) has been reported to be more glutamine addicted than other breast cancer subtypes. At which stage of the breast tumorigenic process the role of these non-coding interacting codes could preferentially be exerted, is still underexplored. One of the unmet clinical needs in daily cancer treatment is represented by the relapse of the primary tumor either locally or at distant sites. Growing evidence document that frequently distant metastasis and matched primary tumors including TNBC exhibit a rather similar mutational landscape. These findings imply that additional non-genetic alterations might contribute the to the establishment of the metastatic phenotype. Among them, glutamine addiction fuelled by aberrant non-coding RNAs network as that involving circPVT1/miR-33a-5p which unleashes c-MYC-GLS1 transcriptional axis might play an important role. While the pivotal role of c-MYC in cancer metabolic alterations has been firmly established, the mechanistic events governing its aberrant activity have not been fully explored. Herein, our findings identify an upstream post-translational network leading to MYC accumulation and transcriptional activity.

There is growing evidence on the therapeutic potential of inhibiting glutamine metabolism to treat human cancers. This attempt has been so far quite challenging. Indeed, the use of specific GLS1 inhibitors has been very successful in preclinical cellular systems while it has been poorly efficacious in mouse models of pancreatic cancer [30]. Recent evidence documents that broad inhibition of glutamine metabolism rather than inhibition of glutaminolysis might hold higher therapeutic potential. 6-diazo-5-oxo-L-norleucine (DON) has anticancer effects but its therapeutic application has been hampered by gastrointestinal toxicity [30]. Recently, DON has been shown to impair the growth of both primary and metastatic PDA in PDOs and transplantable models. We found that the GLS1 inhibitor BPTES exerted a dose-dependent anti-tumoral activity on PDOs derived from three TNBC metastatic patients (Fig. 6F). These findings highlight the therapeutic potential of GLS1 inhibitors in human cancers with altered glutamine metabolism. Intriguingly, a first-in human biomarker-driven Phase I trial (NCT03894540) testing selective inhibitors of GLS1 (IACS-6274) in patients with solid tumors has been concluded. The results of the trial document that IACS-6274 is well tolerated, with good PK, significant target modulation and preliminary anti-tumoral activity [31]. Indeed, the advantage of the use of specific GLS1 inhibitors resides on their ability to impair directly the first enzyme involved in the glutaminolitic pathway. This effect may also limit GLS1 inhibitor off targets effects that could be present following a broader inhibition of glutamine metabolism.

## Conclusions

Altogether our findings document that the choice of the therapeutic targeting of either glutaminolysis or glutamine metabolism might be related to tumor type, to its stage as primitive tumor or metastatic disease, and to previously administered cancer treatments. Indeed, it would be interesting to investigate if circPVT1 oncogenic role on cancer metabolism is only specific for TNBC or may have a broader impact including other breast cancer subtypes and tumor types. It is still uncertain at which stage of a stepwise tumorigenic process aberrant GLS1 activity takes place. The elucidation of temporal and spatial GLS1 activity might contribute to design combinatorial treatment targeting broadly glutamine metabolism. As plasticity and redundancy represent two key features of cancer metabolism, the therapeutic potential of targeting of the circPVT1/miR-33a-5p/Myc/GLS1 axis requires further experimental validation.

## Electronic supplementary material

Below is the link to the electronic supplementary material.

Supplementary Material 1

Supplementary Material 2

Supplementary Material 3

Supplementary Material 4

## Data Availability

No datasets were generated or analysed during the current study.

## Abbreviations

BC: Breast cancer
ChIP: Chromatin immunoprecipitation
circRNA: Circular RNAs
DON: 6-diazo-5-oxo-L-norleucine
ER: Estrogen receptor
GLS1: Glutaminase 1
GLUD1: Glutamate dehydrogenase 1
HER2: Epidermal Growth factor receptor 2
lncRNA: Long non-coding RNAs
miRNAs: Micro RNAs
PDO: Patient derived organoid
PK: Pharmacokinetics
PR: Progesterone receptor
TCA: Tricarboxylic acid cycle
TCGA: The Cancer Genome Atlas
TNBC: Triple negative breast cancer
