# Supplementary material for: CircPVT1 weakens miR-33a-5p unleashing the c-MYC/GLS1 metabolic axis in breast cancer
Source: J Exp Clin Cancer Res. 2025 Mar 20;44:100. doi: 10.1186/s13046-025-03355-1 (PMC11924866; doi:10.1186/s13046-025-03355-1)
Supplement: Supplementary file 1 — Supplementary Material 1 [file 13046_2025_3355_MOESM1_ESM.docx]

**
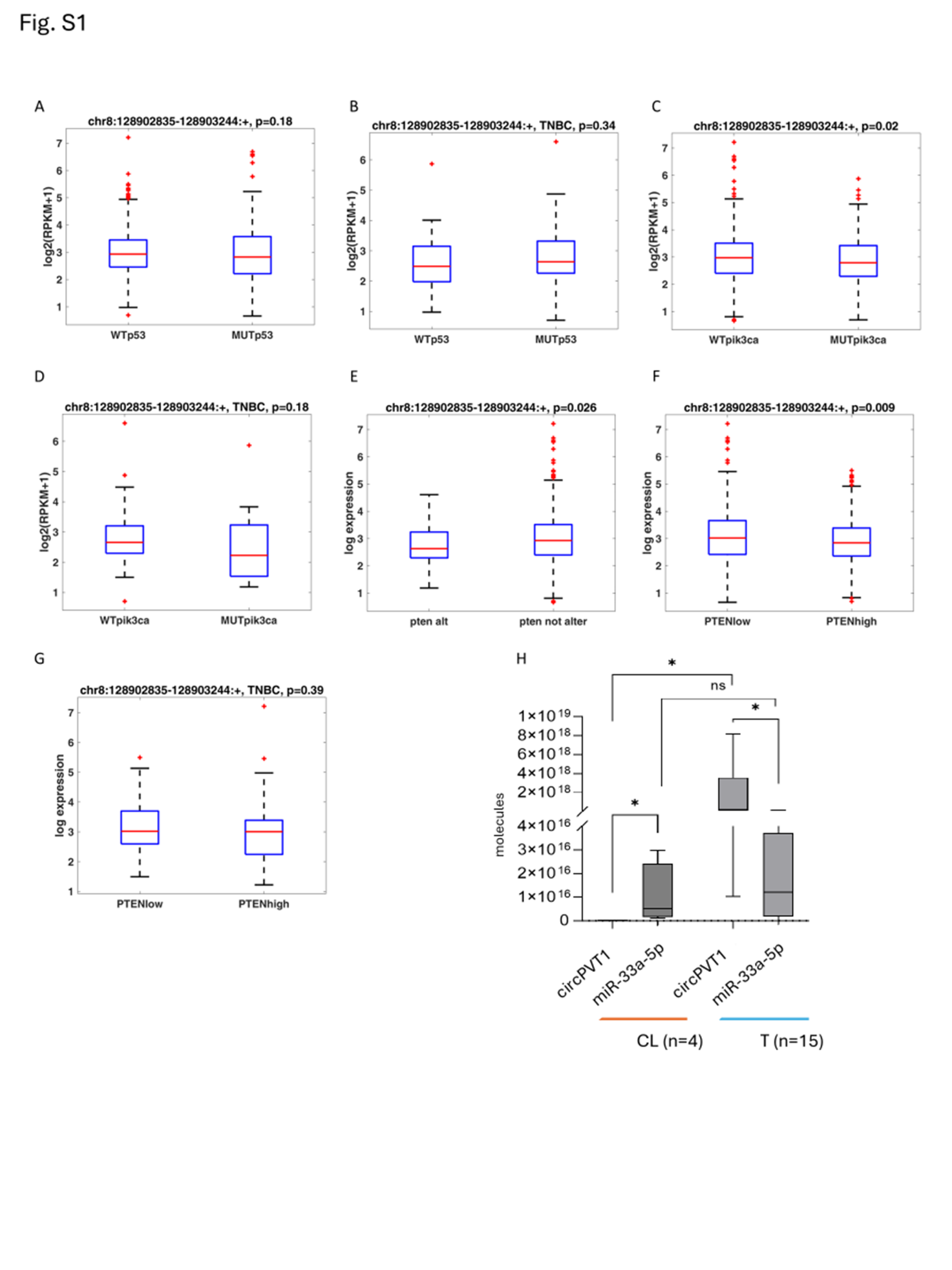
**

**Fig.S1** (A-D) Box plots show the association between circPVT1 expression and the mutation of either *TP53* (A-B) or *PIK3CA* (C-D) from TCGA breast cancer and TNBC data set. (E-G) Box plots show the association between circPVT1 expression and PTEN genomic alteration (E) and its expression levels (F-G) from TCGA breast cancer (F) and TNBC (G) data set. All statistical differences were assessed by Wilcoxon test. (H) Number of circPVT1 and miR-33a-5p molecules per samples in 15 TNBC tissue samples and in 4 contralateral ones.
