# Supplementary material for: CircPVT1 weakens miR-33a-5p unleashing the c-MYC/GLS1 metabolic axis in breast cancer
Source: J Exp Clin Cancer Res. 2025 Mar 20;44:100. doi: 10.1186/s13046-025-03355-1 (PMC11924866; doi:10.1186/s13046-025-03355-1)
Supplement: Supplementary file 2 — Supplementary Material 2 [file 13046_2025_3355_MOESM2_ESM.docx]

**
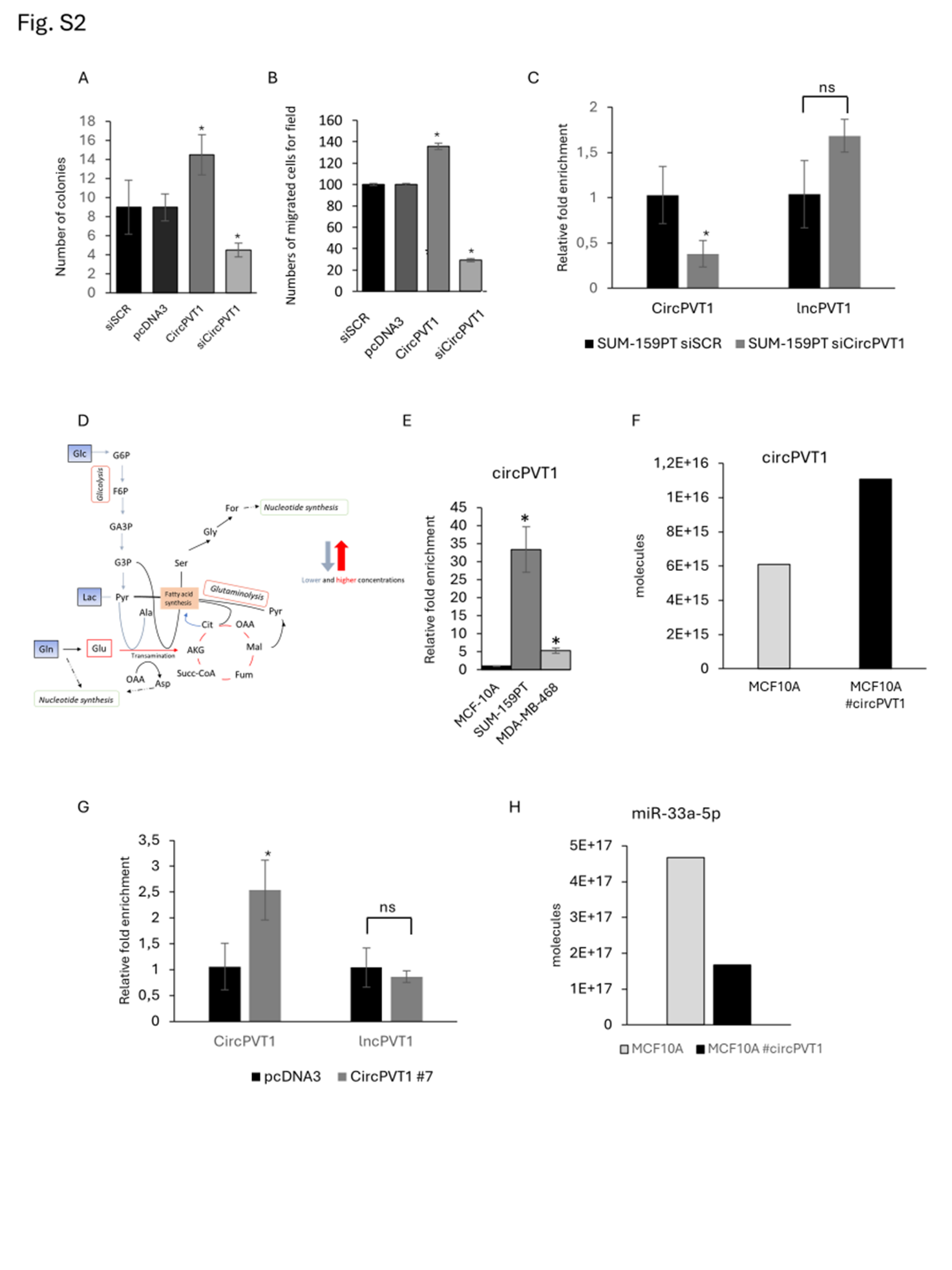
**

**Fig.S2** (A) Histograms show the number of colonies of MDA-MB-468 cells either expressing high or low levels of circPVT1. (B) Histograms show the number of migrated MDA-MB-468 cells treated as in A. (C) Histograms show the levels of lncPVT1 expression in SUM-159PT silenced for circPVT1. (D) Metabolic pathway influenced by circPVT1 silencing in SUM-159PT cells. (E) Histograms show circPVT1 expression levels in MCF-10A, SUM-159PT and MDA-MB-468 cells. (F) Number of circPVT1 molecules per samples (one million cells) in MCF-10A overexpressing circPVT1 compared to the control MCF-10A. (G) Histograms show the levels of lncPVT1 expression in MCF-10A cells overexpressing circPVT1. (H) Number of miR-33a-5p molecules per samples (one million cells) in MCF-10A overexpressing circPVT1 respect to the control MCF-10A.
