# Supplementary material for: CircPVT1 weakens miR-33a-5p unleashing the c-MYC/GLS1 metabolic axis in breast cancer
Source: J Exp Clin Cancer Res. 2025 Mar 20;44:100. doi: 10.1186/s13046-025-03355-1 (PMC11924866; doi:10.1186/s13046-025-03355-1)
Supplement: Supplementary file 3 — Supplementary Material 3 [file 13046_2025_3355_MOESM3_ESM.docx]

**
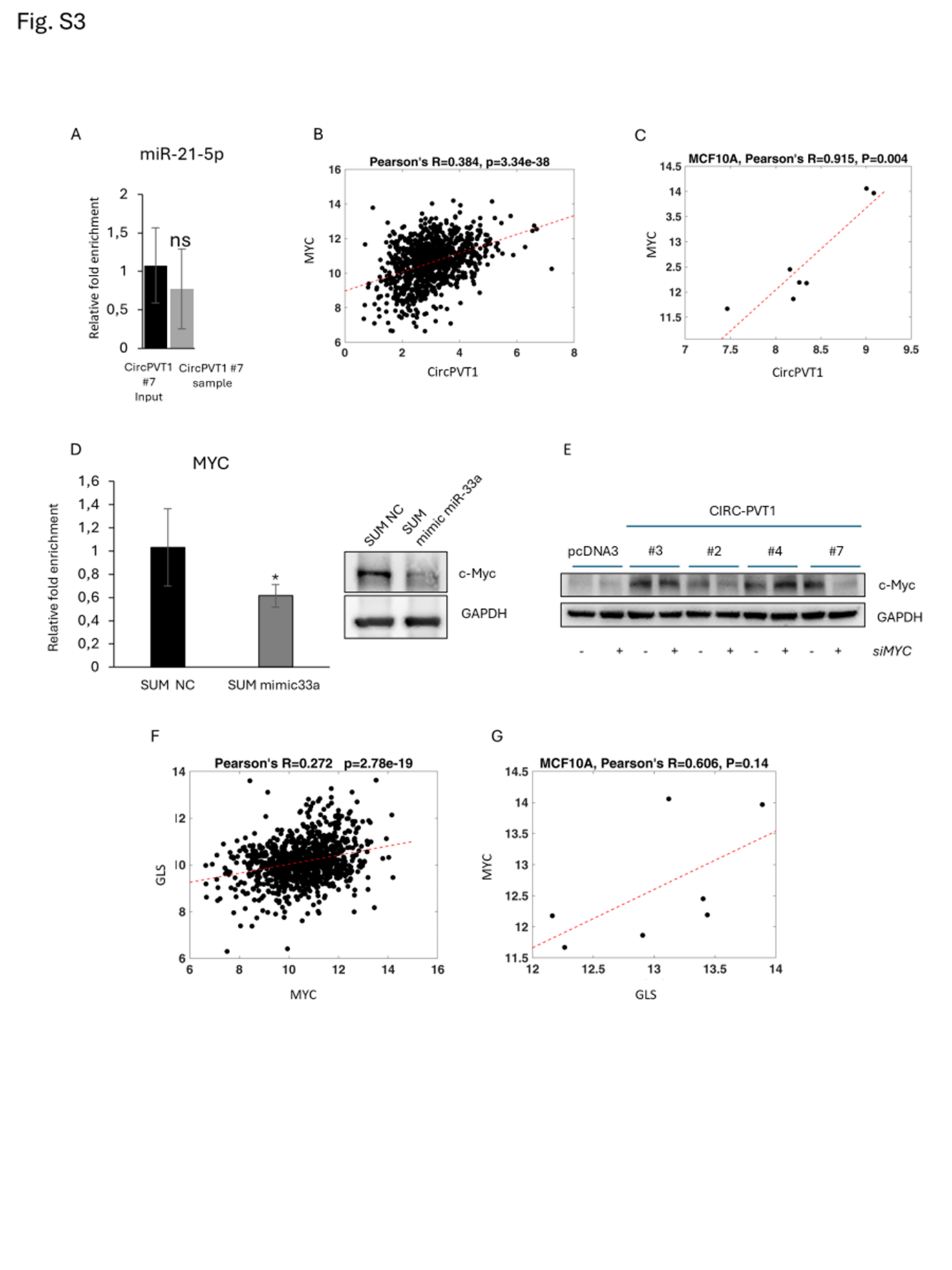
**

**Fig.S3** (A) Histograms show the miR-21-5p relative fold enrichment in MCF-10 circPVT1#7 cells measured in total RNA immunoprecipitated with circPVT1-capture probes. (B-C) Pearson positive correlation between c-MYC and circPVT1 expression levels from TCGA breast data set (B) or from seven different clones of MCF-10A ectopically expressing high levels of circPVT1 (C). (D) (left panel) Histograms show the expression level of c-Myc after overexpression of miR-33a-5p and (right panel) relative protein gel blot of cell lysates obtained from SUM-159PT after overexpression of mimic miR-33a-5p. (E) Uncropped protein gel blot of whole cell lysates extracted from MCF-10A cells depleted or not for c-Myc expression reported in Fig. 4F. (F) Pearson positive correlation between c-MYC and GLS expression levels from TCGA breast data set (F) or from seven different clones of MCF-10A ectopically expressing high levels of circPVT1 (G).
