# Supplementary material for: CircPVT1 weakens miR-33a-5p unleashing the c-MYC/GLS1 metabolic axis in breast cancer
Source: J Exp Clin Cancer Res. 2025 Mar 20;44:100. doi: 10.1186/s13046-025-03355-1 (PMC11924866; doi:10.1186/s13046-025-03355-1)
Supplement: Supplementary file 4 — Supplementary Material 4 [file 13046_2025_3355_MOESM4_ESM.docx]

**
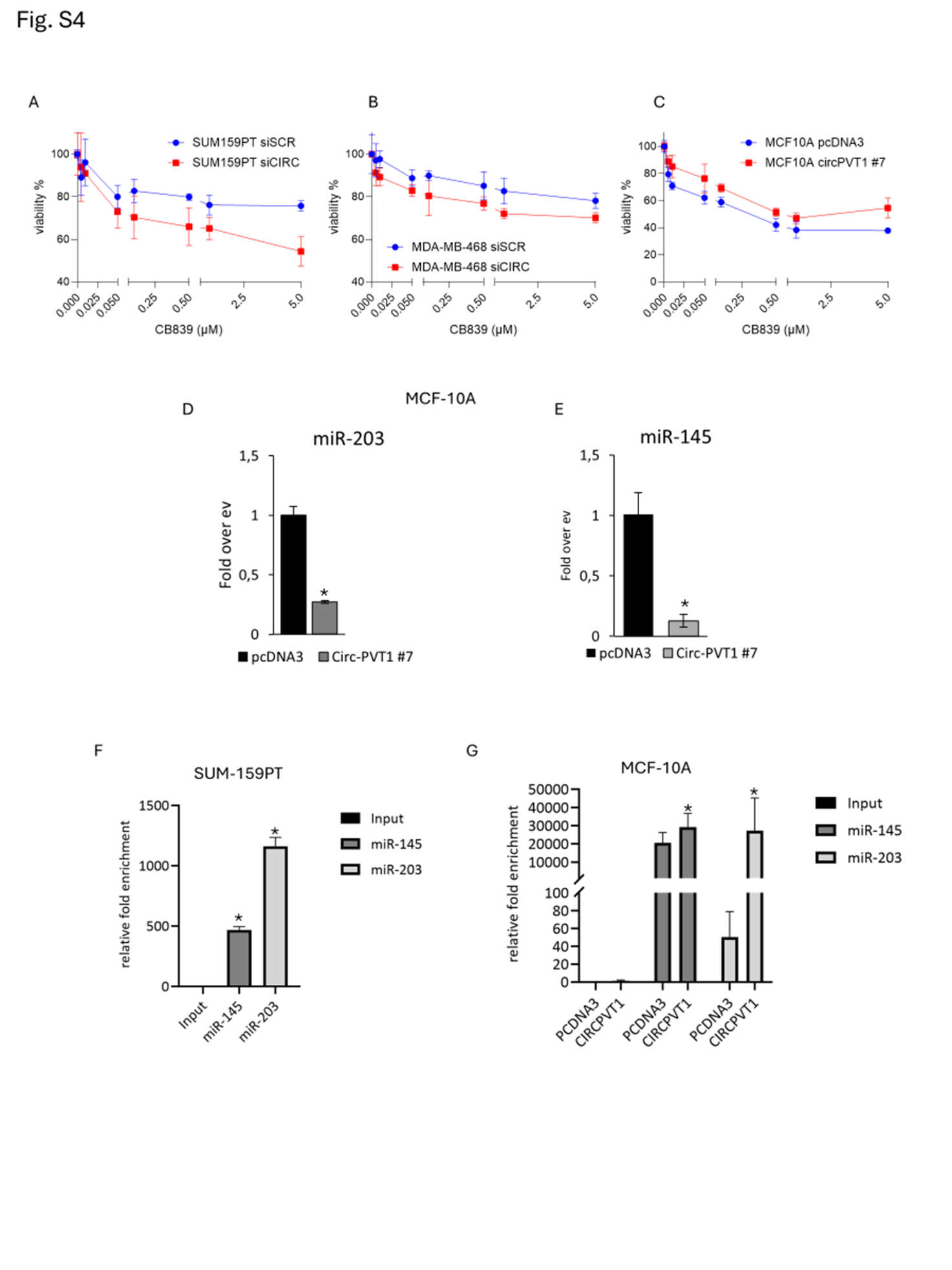
**

**Fig. S4** (A) Viability curves obtained by measuring ATP levels in SUM-159PT cells silenced or not for circPVT1 and treated for 72 hrs with increasing doses of CB839 (0 – 5 µM). (B) Viability curves obtained by measuring ATP levels in MDA-MB-468 cells silenced or not for circPVT1 and treated for 72 hrs with increasing doses of CB839 (0 – 5 µM). (C) Viability curves obtained by measuring ATP levels in MCF-10A circPVT1#7 and treated for 72 hrs with increasing doses of CB839 (0 – 5 µM). (D-E) Histograms show the expression levels of miR-203 (D) and miR-145 (E) measured in MCF-10A circPVT1#7. (F-G) Histograms show the miR-203 and miR-145 relative fold enrichment in SUM-159PT (F) and MCF-10 circPVT1#7 (G) cells measured in total RNA immunoprecipitated with circPVT1-capture probes.
